# Highly Sensitive Impact Sensor Based on PVDF-TrFE/Nano-ZnO Composite Thin Film

**DOI:** 10.3390/s19040830

**Published:** 2019-02-18

**Authors:** Jing Han, Dong Li, Chunmao Zhao, Xiaoyan Wang, Jie Li, Xinzhe Wu

**Affiliations:** 1 College of Mechatronic Engineering, North University of China, Taiyuan 030051, China; lidongnuc@sina.com (D.L.); wdayanyan@sina.com (X.W.); wxznuc@sina.com (X.W.); 2 School of Materials Science and Engineering, North University of China, Taiyuan 030051, China; zhaochunmaonuc@sina.com

**Keywords:** piezoelectric, PVDF-TrFE, nano-ZnO, flexion sensor

## Abstract

A thin film of polyvinylidene fluoride-trifluoroethylene (PVDF-TrFE) has good flexibility and simple preparation process. More importantly, compared with PVDF, its piezoelectric β-phase can be easily formed without mechanical stretching. However, its piezoelectricity is relatively lower. Therefore, at present, PVDF-TrFE is always compounded with other kinds of piezoelectric materials to solve this problem. The effect of nano-ZnO doping amount on the sensing characteristics of the piezoelectric films was studied. PVDF-TrFE/nano-ZnO films with different nano-ZnO contents were prepared by spin coating process and packaged. The dispersion of nano-ZnO dopants and the crystallinity of β-phase in piezoelectric films with different nano-ZnO contents were observed by scanning electron microscopy and X-ray diffraction, and the piezoelectric strain constants and dielectric constants were measured, respectively. The effect of different nano-ZnO contents on the output performance of the piezoelectric sensor was obtained by a series of impact experiments. The results show that the piezoelectric strain constant and dielectric constant can be increased by doping nano-ZnO in PVDF-TrFE. Moreover, the doping amount of nano-ZnO in PVDF-TrFE is of great significance for improving the piezoelectric properties of PVDF-TrFE/nano-ZnO thin films. Among the prepared piezoelectric films, the output voltage of PVDF-TrFE/nano-ZnO piezoelectric sensor with 7.5% nano-ZnO doping amount is about 5.5 times that of pure PVDF-TrFE. Thus, the optimal range of the doping amount for nano-ZnO is about 4–10%.

## 1. Introduction

In recent years, more and more studies have focused on organic piezoelectric materials such as polyvinylidene fluoride (PVDF) series and its copolymer polyvinylidene fluoride-trifluoroethylene (PVDF-TrFE), because of their flexibility, low cost, and stable performance. At present, they have been widely adapted for various applications, such as ultrasonic measurement [1,2,3,4,5], pressure sensing [6,7,8,9,10], energy harvesting [11,12,13,14,15,16,17] and wearable devices [18,19,20,21]. Compared with PVDF, PVDF-TrFE forms a piezoelectric β-phase directly from the melt-crystallization process without mechanical stretching, which simplifies the material preparation process and greatly improves the crystallinity of the piezoelectric copolymer. To date, however, the piezoelectric performance of pure PVDF-TrFE is about 15–20 pC/N, which is significantly lower than that of other composite piezoelectric thin film [22,23], and has become the biggest obstacle for its wide practical applications.

Nano-ZnO is a good piezoelectric material with strong voltage and stable chemical properties. Due to its unique effects on the surface, volume, and quantum, as well as dielectric confinement, it exhibits excellent performance in catalysis, electricity and mechanics, and has important applications in many fields such as chemical, electronics and ceramics. Therefore, to enhance the piezoelectric and material mechanical performance, some researchers have begun to study the PVDF-based composite technology by embedding nano-ZnO particles.

With regard to composite architecture, the microstructure and electromechanical properties of PVDF-based piezoelectric films are greatly affected by the size and surface functional state of ZnO nanoparticles. Specifically, nano-ZnO has the natural properties with small size, large specific surface area, different bond states on the surface and the bond states inside the particles, as well as incomplete coordination of surface atoms. The surface active sites are increased, forming uneven atomic steps and expanding the contact surface. With the decrease of ZnO nanoparticle size, the energy storage level of piezoelectric film increases linearly. However, it has less influence on the crystallinity of PVDF-TrFE [24]. Meanwhile, there are many nano-morphologies for nano-ZnO, such as nanotubes, nanorods and nanowires. By optimizing the ZnO nanostructure and spatial layout, the performance of composites is higher than that of pure PVDF [25]. On the other hand, various forms of nano-ZnO lead to different static friction and surface characteristics. All these will undoubtedly affect the arrangement of PVDF-TrFE chains, resulting in obtaining different crystallinity and piezoelectric properties [26]. In addition, the interaction between the surface charge of ZnO nanorods [27] or nanofibers [28] and the dipole is beneficial to increase the β-phase content and the piezoelectric output.

However, although the above-mentioned studies have proved that the piezoelectric property of PVDF-TrFE can be improved by composting nano-ZnO, the influence of doping content is a another important factor that cannot be neglected. In fact, with the increase of nano-ZnO content, the residual polarization and piezoelectric property of PVDF-TrFE/ZnO will be improved simultaneously, but the breakdown voltage is reduced [29]. On the other hand, with increasing of doping amount, the agglomeration of ZnO nanoparticles will be intensified, which directly effects the total quality of the film. Thus, we report a surface modification method to improve the dispersion of ZnO by adding a dispersant (n-propylamine, PA) and a silane coupling agent [30]. The modified ZnO was beneficial to the piezoelectric outputs of thin film. The optimal amount of doping is not given.

The effect of different nano-ZnO contents on the output performance of composite thin film was studied by using the proposed method. To measure the impact energy, a PVDF-TrFE/ZnO sensor device was completed and its output performance was obtained by a series of impact experiments. It was found that, in contrast with other impact sensors, the sensitivity of our sensor can be improved and the doping amount of ZnO can be reduced.

## 2. Experiment

### 2.1. PVDF-TrFE/Nano-ZnO Film Preparation

**Figure 1:**
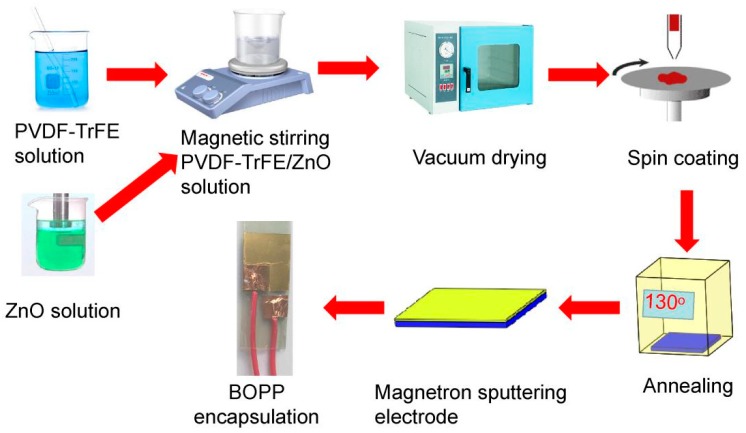
Flow chart of PVDF-TrFE/nano-ZnO piezoelectric film preparation.

PVDF-TrFE/nano-ZnO films with different nano-ZnO contents were prepared by spin coating. PVDF-TrFE powder was obtained from Wuhan Cymenes LLC (Wuhan, China). Acetone and N,N-dimethylformamide were provided by Tianjin Chemical Reagent Red Rock. Nano-ZnO particles (particle size = 30 ± 10 nm, Mw = 81.37) were purchased from Aladdin Industrial Corporation. The flexible substrate Indium tin oxide-Polyethylene naphthalate (ITO-PEN) was obtained from Southern China Xiangcheng Technology Co., Ltd., Shenzhen, China. The type of vacuum drying oven is TAISITE DZ-1BC. The spin coating equipment is a KW-4A type glue homogenizing machine produced by the Institute of Microelectronics, Chinese Academy of Sciences. The PVDF-TrFE/nano-ZnO film preparation process is shown in Figure 1. The specific steps are as follows:

#### 2.1.1. Prepare PVDF-TrFE/Nano-ZnO Composite Solution

At room temperature, the PVDF-TrFE powder and N,N-Dimethylformamide were mechanically mixed for 60 min to obtain a transparent viscous clarified PVDF-TrFE solution; nano-ZnO with a particle size of about 30 nm. Acetone, n-Propylamine (PA) and 1H, 2H, 1H, 2H, perfluoroalkyltriethoxysilanes (PFOES) were mixed by ultrasonic stirring at room temperature for 30 min, and a transparent and clarified nano-ZnO dispersion was obtained. The nanometer ZnO dispersion was injected into the PVDF-TrFE solution with a mass fraction of 0%, 1.5%, 4.5%, 7.5%, 10.5% or 12.5%. It was then magnetically stirred to obtain translucent homogeneous PVDF-TrFE/nano-ZnO solution with different nano-ZnO content.

#### 2.1.2. Prepare PVDF-TrFE/Nano-ZnO Composite Film

The semitransparent and homogeneous PVDF-TrFE/nano-ZnO solution was placed in the vacuum drying oven, and allowed to stand at a vacuum of 0.09 MPa for 30 min at room temperature to removed bubbles in the solution. The clean ITO-PEN flexible substrate was fixed to the homogenizer, and the transparent and uniform solution was dripped into the center of the flexible substrate by using the pipette and rotated at 500 r/min for 18 s and then at 2500 r/min speed for 60 s. After the homogenizer stopped rotating, the semi-transparent homogeneous solution was again dropped into the center of the ITO-PEN flexible substrate, and the rotation step was repeated to obtain a composite film with five layers. Then, the flexible substrate with a composite film was placed in a vacuum drying box under the 0.09 MPa vacuum. After heating up to 130 °C, the films were annealed at the constant temperature for 1 h to improve quality of the films.

#### 2.1.3. Prepare Piezoelectric Sensors

A gold electrode was sputtered over the film by magnetron sputtering, and the ITO attachment layer of the ITO-PEN flexible substrate was used as the lower electrode. The pins were staggered on the upper and lower electrodes, and the electrodes were extracted using copper tape adhesive wires. Finally, Biaxially Oriented Polypropylene (BOPP) film was used to encapsulate it and a measurable piezoelectric sensor was obtained.

**Figure 2:**
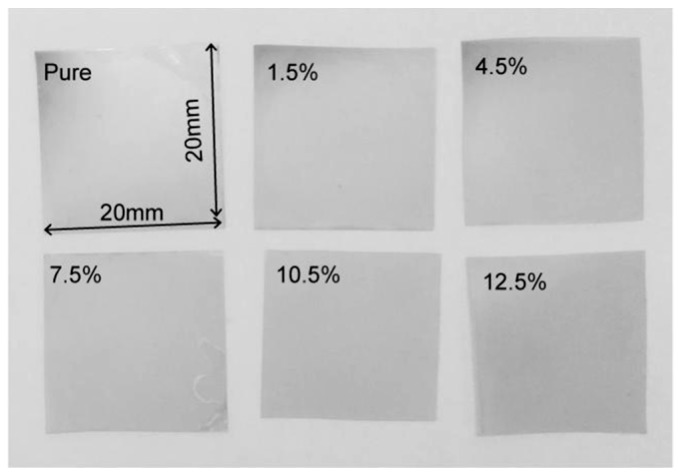
Six kinds of PVDF-TrFE/nano-ZnO piezoelectric films with different nano-ZnO contents were prepared.

**Figure 3:**
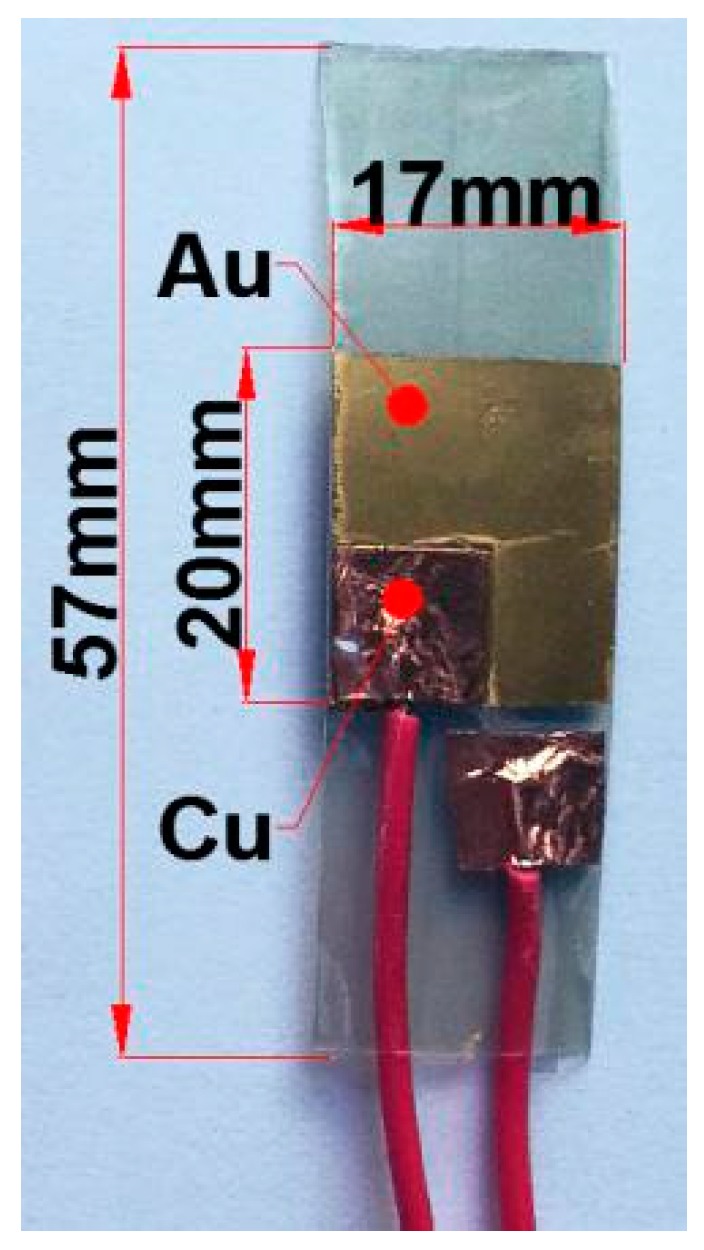
PVDF-TrFE/nano-ZnO piezoelectric sensor with 7.5% nano-ZnO contents has been prepared.

**Figure 4:**
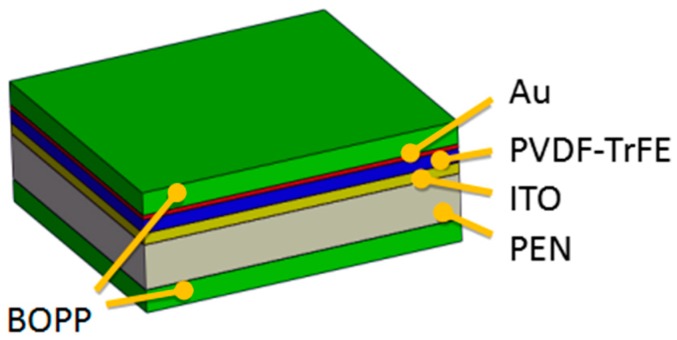
Cross-sectional structure diagram of PVDF-TrFE/nano-ZnO film sensor.

After the above steps, the pure PVDF-TrFE piezoelectric film and the PVDF-TrFE/nano-ZnO piezoelectric films with 1.5%, 4.5%, 7.5%, 10.5%, and 12.5% nano-ZnO doping, respectively, were prepared as shown in Figure 2, and the size of the prepared film was 20 mm × 20 mm. The finished PVDF-TrFE/nano-ZnO piezoelectric sensors with 7.5% nano-ZnO doping are shown in Figure 3, and the effective size was 20 mm × 17 mm. Figure 4 shows the cross-sectional structure schematic of the PVDF-TrFE/nano-ZnO piezoelectric sensor based on the actual structure.

### 2.2. Piezoelectric Film Performance Test

To study the dispersion of nano-ZnO in the prepared PVDF-TrFE/nano-ZnO piezoelectric film, at 30 kV operating voltage and 50 µm scale, the dopant dispersion of PVDF-TrFE/nano-ZnO piezoelectric films with different nano-ZnO contents was recorded by JEOLJSM-7001F scanning electron microscope (JEOL, Tokyo, Japan).

The β-phase is the crystal phase that contributes most to the piezoelectric properties of PVDF-TrFE film. The β-phase content of PVDF-TrFE/nano-ZnO piezoelectric films with different nano-ZnO dopants was measured by D/MAX-RB X-ray diffractometer, and the effect of nano-ZnO dopants on the β-phase was studied.

Piezoelectric strain constant is an important parameter reflecting the properties of piezoelectric materials. In this paper, using the ZJ-3AN quasi-static piezoelectric strain constant d_33_ measuring instrument produced by the Institute of Acoustics of the Chinese Academy of Sciences, the piezoelectric strain constant d_33_ of the obtained film was tested. Each film was measured five times, and the average value was taken as the piezoelectric strain constant.

The dielectric constant reflects the ability of material to maintain charge, which is especially important for piezoelectric materials. The PVDF-TrFE/nano-ZnO piezoelectric film was measured using an Agilent 4294A precision impedance analyzer and a matching 16034E fixture. The modulus and phase difference of the impedance were measured as a function of frequency. The electrical impedance of the piezoelectric film can be expressed as:

(1) Z=R+jX=|Z|(cosψ+jsinψ)

where $\left|Z\right|=V_{T}/I_{T}$ is the impedance module, *ψ* is the phase difference between current and voltage, and *R* and *X* are the real and imaginary parts of the electrical impedance at a given frequency *f*, respectively.

The relationship between capacitance and impedance of piezoelectric thin film can be expressed as follows:

(2) $$C=-\frac{1}{2\Pi f}(\frac{X}{R^{2}+X^{2}})$$

The dielectric constant of PVDF-TrFE/nano-ZnO piezoelectric film can be obtained from the capacitance and its geometric parameters.

(3) $$\varepsilon =\frac{hC}{S}$$

(4) $$\varepsilon_{r}=\frac{\varepsilon }{\varepsilon_{0}}$$

where *ε* is the dielectric constant of the PVDF film, *S* is the end surface area, and *h* is the thickness of the piezoelectric film, *ε*_0_ = 8.85 × 10^−12^ *C*^2^/(N·M^2^) is the vacuum dielectric constant.

According to the above formula, the relationship between the dielectric permittivity and the frequency can be obtained.

### 2.3. Performance Test of Piezoelectric Sensor

**Figure 5:**
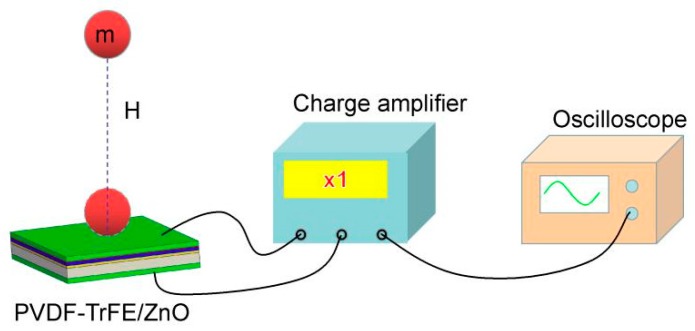
Schematic diagram of impact experiment.

For a better understanding of the difference of piezoelectric properties, the piezoelectric output properties of PVDF-TrFE/nano-ZnO film sensors with different nano-ZnO content in the actual environment were tested by impact experiments. Using a 25 mm diameter steel ball (mass of 65.0 g), which was dropped at different heights, as the input energy of the PVDF-TrFE/nano-ZnO piezoelectric sensor, the voltage signal generated by the sensor was amplified by one times the charge amplifier (Yangzhou KD5007, Kedong Electronic Co., Ltd., Yangzhou, China); the charge signal was converted into a voltage signal; and the output signal of the sensor was recorded by a digital storage oscilloscope (ISDS205B, Instrustar Electronic Technology Co., Ltd., Harbin, China). Five experiments were performed at each height, and the average value was taken as the output signal under different impact energy. The experimental test device is shown in Figure 5.

## 3. Results and Discussion

### 3.1. Film Microstructure

**Figure 6:**
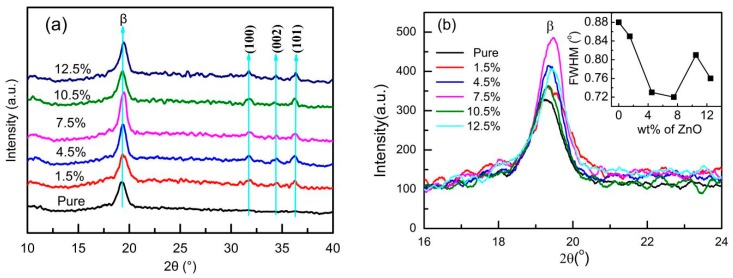
WXRD spectra of different nano-ZnO doped PVDF-TrFE/nano-ZnO films: (**a**) XRD patterns; and (**b**) peak intensity of β-phase and FWHM of β-phase with different ZnO Contents.

Figure 6a shows the WXRD spectra of PVDF-TrFE/nano-ZnO films with various nano-ZnO doping amounts. It can be seen that pure PVDF-TrFE only contains the diffraction peak of 2θ = 19.6 degrees, which is the characteristic peak of β-phase. The WXRD spectra of other PVDF-TrFE/nano-ZnO films show diffraction peaks at 31.9 degrees (100), 34.5 degrees (002) and 36.3 degrees (101), all of which are characteristic peaks of nano-ZnO. Figure 6b shows the peak intensity and full width at half maximum (FWHM) of the β-phase at different nano-ZnO contents. When the nano-ZnO doping amount was 1.5%, 4.5%, 7.5%, 10.5%, and 12.5%, the peak intensity was 336.0, 350.4, 454.6, 298.8 and 379.6, respectively. The corresponding FWHM was 0.85, 0.73, 0.72, 0.81 and 0.76. Among them, the peak intensity value located at the 19.6-degree was the largest, and the FWHM was the smallest, indicating that the maximum β-phase content was obtained.

**Figure 7:**
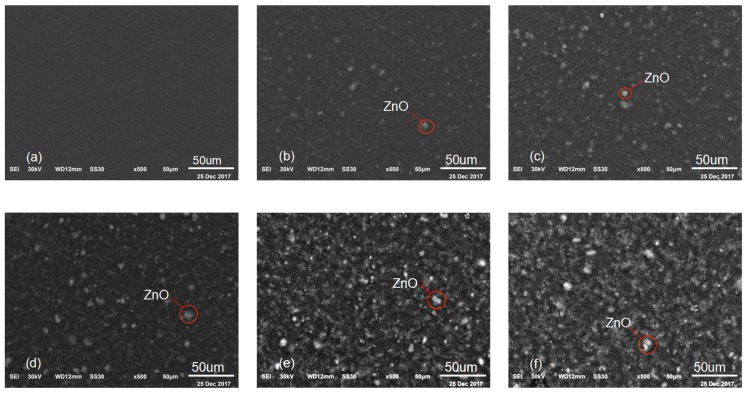
Scanning Electron Microscopy of PVDF-TrFE/nano-ZnO films with different nano-ZnO contents at 50 uµ: (**a**) micromorphology of pure PVDF-TrFE; (**b**) dispersion of nano-ZnO in PVDF-TrFE/nano-ZnO films with 1.5% nano-ZnO content; (**c**) nano-ZnO dispersion diagram of film with 4.5% nano-ZnO content; (**d**) nano-ZnO dispersion diagram of film with 7.5% nano-ZnO content; (**e**) nano-ZnO dispersion diagram of film with 10.5% nano-ZnO content; and (**f**) nano-ZnO dispersion diagram of film with 12.5% nano-ZnO content.

Figure 7 shows the dispersion of nano-ZnO dopants by scanning electron microscope of PVDF-TrFE/nano-ZnO films with different nano-ZnO contents at 50 µm scale. Figure 7a shows the microscopic morphology of pure PVDF-TrFE. Figure 7b–f shows the nano-ZnO dopants dispersion of PVDF-TrFE/nano-ZnO with nano-ZnO content 1.5%, 4.5%, 7.5%, 10.5% and 12.5%, respectively. It can clearly be seen that, with the increase of nano-ZnO content, the clustering part of nano-ZnO gradually increased. The reason is that there are many suspension bonds and unsaturated bonds on the surface of ZnO nanoparticles; they have high activity and instability; and they are easy to combine with other atoms and tend to be stable. Meanwhile, the dispersion of nano-ZnO can be improved significantly by using of the surface modification method. According to our previous study, the distribution of nano-ZnO in the PVDF-TrFE/nano-ZnO film with modifier PA and a silane coupling agent is more uniform and the dispersion is better [30].

### 3.2. Piezoelectric Strain Constant d_33_

**Figure 8:**
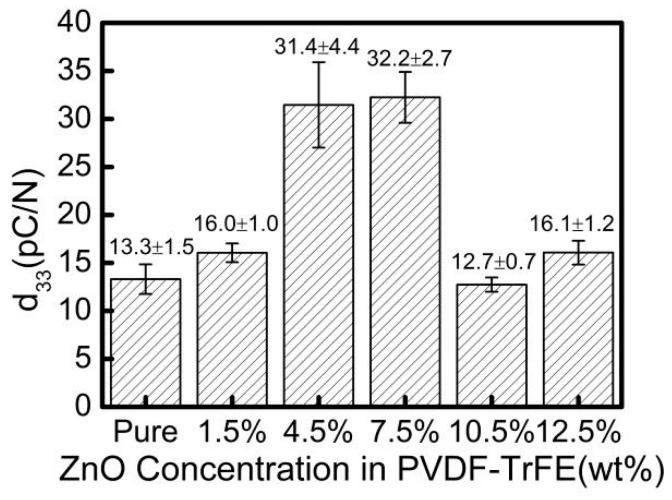
Piezoelectric strain constant d_33_ of the PVDF-TrFE/nano-ZnO films with different nano-ZnO contents.

The piezoelectric strain constant d_33_ of the PVDF-TrFE/nano-ZnO piezoelectric film with different nano-ZnO content was measured, as shown in Figure 8. The addition of nano-ZnO into PVDF-TrFE could increase the piezoelectric strain constant of the film. When the ZnO content was 7.5%, the piezoelectric strain constant d_33_ of the PVDF-TrFE/nano-ZnO film was the largest, 142% higher than that of the pure PVDF-TrFE. These changes in value of d_33_ could be attributed to three factors. Firstly, from the internal performance mechanism of the film, the interaction between nano-ZnO surface charge and PVDF-TrFE dipole could improve the crystallinity of β-phase [25], and the influence of static friction between nano-ZnO particles and PVDF-TrFE particles on the chain arrangement of PVDF-TrFE could also promote the formation of β-phase [29]. This conclusion can be confirmed by the WXRD spectrum in Figure 6. Secondly, according to the principle of piezoelectric effect, under the action of external force, the PVDF-TrFE/nano-ZnO piezoelectric film generates a potential field and distributes it along the longitudinal direction. Because the dielectric properties of PVDF-TrFE and nano-ZnO were different, the distribution of potential in the film was not uniform. Therefore, a lateral additional potential field was generated between PVDF-TrFE and nano-ZnO, which played a role in increasing the piezoelectric strain constant d_33_ [31]. Thirdly, under the action of external forces, because the elastic moduli of the PVDF-TrFE and nano-ZnO materials were different, the strain of the two materials was different, which also led to the existence of additional stress inside, improving the detection of the piezoelectric output, that is, increasing the piezoelectric strain constant.

However, the relationship between nano-ZnO content and piezoelectric strain constant d_33_ was not simply proportional. An increase of ZnO content to 10.5% rapidly decreased the piezoelectric strain constant d_33_ value to ~12.7. This sharp drop of piezoelectric strain constant could be ascribed to agglomeration of nano-ZnO. With the increase of the doping concentration, the dispersion of the non-composite became worse. When the negative effect of dispersion and the positive effect of doping concentration canceled each other, an optimal doping concentration point appeared. According to the WXRD spectra (Figure 6) and the scanning electron micrograph (Figure 7) of piezoelectric films, when the doping amount of nano-ZnO was small, the nano-ZnO was uniformly distributed in the PVDF-TrFE polymer, satisfying the assumption of the Yamada model [32,33], and the β-phase crystallinity increased as the doping content increased. Therefore, when the nano-ZnO doping content was less than 7.5%, the piezoelectric strain constant d_33_ increased with the increase of nano-ZnO doping amount; when the nano-ZnO doping amount was greater than 7.5%, due to the influence of the preparation process and other factors, the phenomenon of agglomeration of nano-ZnO dopant increased, the shape factor changed, and the crystallinity of the piezoelectric film decreased, resulting in the decrease of piezoelectric strain constant of the PVDF-TrFE/nano-ZnO piezoelectric films.

Therefore, doping nano-ZnO in PVDF-TrFE film could improve the piezoelectric strain constant, and, when the amount of nano-ZnO doping was less than 10%, the agglomeration could be controlled.

### 3.3. Dielectric Constant

**Figure 9:**
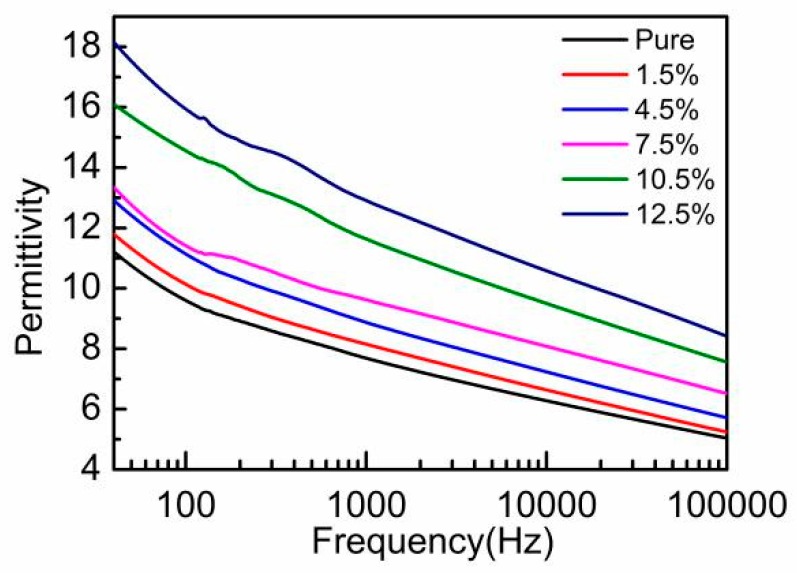
Relative dielectric constant versus frequency curves of PVDF-TrFE/nano-ZnO films with different nano-ZnO contents.

The relationship between the relative permittivity and frequency of PVDF-TrFE/nano-ZnO films with different nano-ZnO contents is shown in Figure 9. The dielectric constant decreased with the increase of frequency. The reason was that, when the applied electric field frequency increased, the charge movement speed was lower than the change speed of high-frequency electric field, so the polarization decreased and the dielectric constant decreased with the increase of frequency.

As the nano-ZnO content increased, the dielectric constant also increased. The polarization of the dielectric mainly included three modes: interface polarization, steering polarization and displacement polarization. The above phenomenon was analyzed from this point of view. First, under the action of the external electric field, because nano-ZnO and PVDF-TrFE had different conductivity, when the charge moved through the conductive phase, the high-resistance phase interrupted the movement, showing that electrons or ions gathered at the interface between nano-ZnO and PVDF-TrFE. The interface polarization was formed, and the overall dielectric performance was enhanced. Second, the increase of nano-ZnO particles also introduced more dipole groups, increased the dipole polarization characteristics, form steering polarization, and increased the dielectric constant. Therefore, doped nano-ZnO could improve the dielectric constant of piezoelectric film.

Piezoelectric strain coefficient d33 can reflect the coupling relationship between elastic properties and dielectric properties of piezoelectric materials. The results in Figure 9 show that, as the nano-ZnO content increased, the dielectric constant also increased. On the other hand, with the increase of the nano-ZnO doping content, the mechanical elasticity of PVDF-TrFE/nano-ZnO film reduced. Therefore, there was a competitive mechanism between mechanical and dielectric properties and, when the doping content of ZnO was 7.5%, as shown in Figure 8, the value of d_33_ reached its maximum.

### 3.4. Output of PVDF-TrFE/Nano-ZnO Sensors

**Figure 10:**
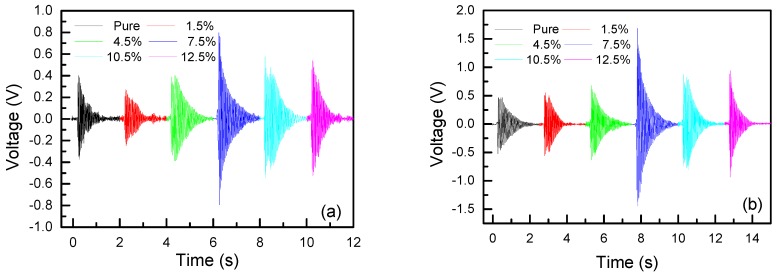

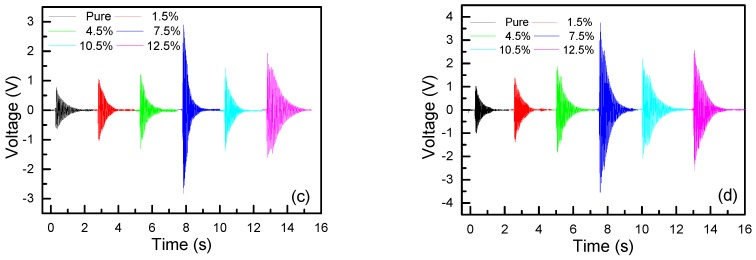
The output signals of PVDF-TrFE/nano-ZnO films with different nano-ZnO contents under different impact energy: (**a**) 54 mJ impact energy; (**b**) 109 mJ impact energy; (**c**) 219 mJ impact energy; and (**d**) 329 mJ impact energy.

**Figure 11:**
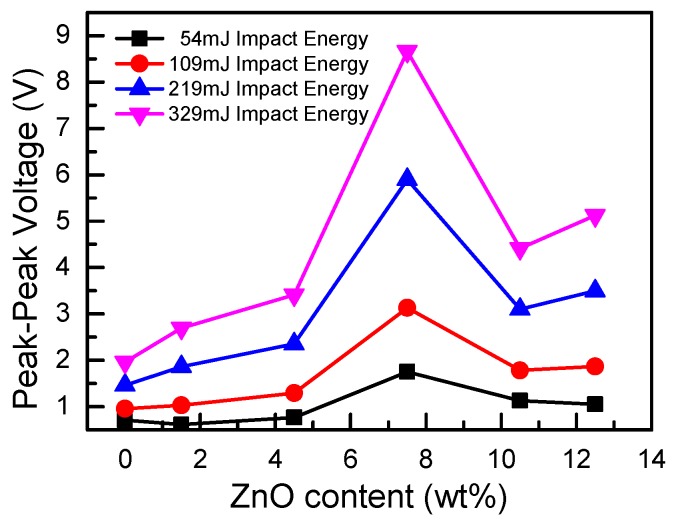
The peak-to-peak voltage versus nano-ZnO dopant under different impact energy.

The output signals of PVDF-TrFE/nano-ZnO sensors with different nano-ZnO contents under different impact energy are shown in Figure 10. The output voltage of the PVDF-TrFE/nano-ZnO piezoelectric sensor was larger than that of the pure PVDF-TrFE piezoelectric sensor under the same impact energy. With the increase of nano-ZnO content, the output voltage increased first and then decreased; for the same sensor, as the impact energy increased, its output performance also increased. The output voltage of different PVDF-TrFE/nano-ZnO piezoelectric sensors changed with nano-ZnO content, as shown in Figure 11. With the increase of nanometer ZnO content, the output performance of PVDF-TrFE/nano-ZnO piezoelectric sensor first increased and then decreased, which is consistent with the rule of basic parameters measured by PVDF-TrFE/nano-ZnO film. When the nano-ZnO content was 7.5%, the output voltage of the PVDF-TrFE/nano-ZnO piezoelectric sensor was about four times that of the pure PVDF-TrFE sensor.

**Figure 12:**
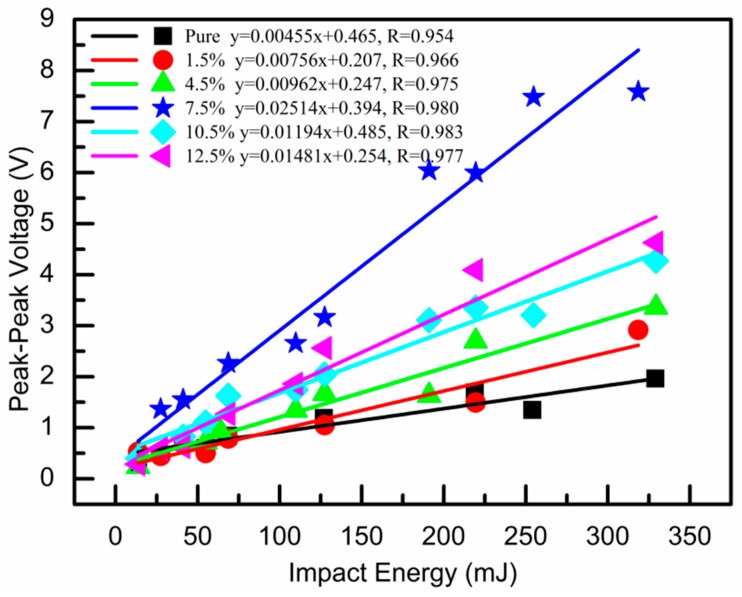
Peak-to-peak voltage versus impact energy curves of PVDF-TrFE/nano-ZnO films with different nano-ZnO contents.

The measured output voltage versus impact energy is shown in Figure 12, where the scatter represents the experimental data, the straight line represents the fitted data, R represents the linear correlation coefficient, and the fitting equation is given. It can be clearly seen that the increase of nano-ZnO content improved the output performance of the piezoelectric sensor and the sensitivity to the impact energy at the same time. The prepared PVDF-TrFE/nano-ZnO piezoelectric sensors had better linearity of output performance and impact energy; all sensors reached above 0.96. According to the fitted linear equation, when the nano-ZnO content was 7.5 %, PVDF-TrFE/nano-ZnO piezoelectric sensor had the maximum impact sensitivity, which was about 0.025 v/mJ.

Table 1 lists the performances of published PVDF-based impact sensors. Here, the equivalent sensitivity, which represents the impact sensitivity of unit sensor area, was used for comparison. It is obvious that the PVDF-TrFE/nano-ZnO sensor with 7.5 wt% ZnO content prepared in this work was much more sensitive than other sensors. The doping amount of ZnO can be decreased to <10%.

## 4. Conclusions

In our work, PVDF-TrFE/nano-ZnO films with nano-ZnO doping content of 1.5%, 4.5%, 7.5%, 10.5%, and 12.5% and pure PVDF-TrFE thin films were prepared by spin-coating method. The effects of nano-ZnO doping amount on the properties of the piezoelectric composites were studied by testing the performance parameters of the composites. When the content of nano-ZnO was less than 10%, it could improve the piezoelectric properties of PVDF-TrFE because of the increase of β-phase crystallinity of PVDF-TrFE. Meanwhile, the piezoelectric strain and dielectric constant of PVDF-TrFE/nano-ZnO also increased accordingly with the increase of doping amount. The agglomeration of nano-ZnO led to a decrease of piezoelectric strain constant. For impact sensor, the output voltage of PVDF-TrFE/nano-ZnO increased with the increase of doping amount and the sensitivity to the impact energy increased gradually. Among the prepared piezoelectric films, the output sensitivity of PVDF-TrFE/nano-ZnO piezoelectric sensor with 7.5% nano-ZnO doping amount was about 5.5 times that of pure PVDF-TrFE.

## Figures and Tables

**Table 1:** Summary of PVDF-based sensors equivalent sensitivity in our study and reported studies.

Sensor		Size (mm)	Equivalent Sensitivity (V/mJ × 10^−3^)	Ref.
Material	ZnO Content (wt%)			
PVDF-TrFE/nano-ZnO	7.5	20 × 17	0.735	This work
PVDF-TrFE/nano-ZnO	20	16.6 × 14.8	0.478	[34]
PVDF-PVP	-	40 × 40	0.652	[35]
PVDF	-	17 × 17	0.654	[36]
